# Integration of breast cancer care in a middle-income country: learning from Suandok Breast Cancer Network (SBCN)

**DOI:** 10.1186/s12885-021-09153-0

**Published:** 2022-01-03

**Authors:** Imjai Chitapanarux, Wimrak Onchan, Panchaporn Wongmaneerung, Areewan Somwangprasert, Nongnuch Bunyoo, Chagkrit Ditsatham, Kirati Watcharachan, Chaiyut Charoentum, Patumrat Sripan, Ausreeya Chumachote, Puttachart Maneesai

**Affiliations:** 1 Suandok Breast Cancer Network, Maharaj Nakorn Chiang Mai Hospital, Faculty of Medicine, Chiang Mai University, Chiang Mai, Thailand; 2 Division of Radiation Oncology, Department of Radiology, Faculty of Medicine, Chiang Mai University, 110 Intawaroros Road, Chiang Mai, 50200 Thailand; 3 Chiang Mai Cancer Registry, Maharaj Nakorn Chiang Mai Hospital, Faculty of Medicine, Chiang Mai University, Chiang Mai, Thailand; 4 Division of Head, Neck, Breast, Department of Surgery, Faculty of Medicine, Chiang Mai University, Chiang Mai, Thailand; 5 Division of Medical Oncology, Department of Internal Medicine, Faculty of Medicine, Chiang Mai University, Chiang Mai, Thailand

**Keywords:** Breast cancer care, Service network, Healthcare access, Overall survival

## Abstract

**Background:**

Breast cancer incidence in Northern Thailand has shown a continuous increase since records began in 1983. In 2002 the urgency of the situation prompted Maharaj Nakorn Chiang Mai Hospital to initiate the Suandok Breast Cancer Network (SBCN).

**Methods:**

The SBCN is a not-for-profit organization in the university hospital which serves as a training and education center and provides highly specialized medical care for patients in Chiang Mai and in 5 provinces of northern Thailand, with the key mission of improving breast cancer care. The short-term goal was to overcome the barriers to engagement with breast cancer and its treatment and the long-term goal was to increase the overall survival rate of breast cancer patients in our region.

**Results:**

We enrolled breast cancer patients treated at Maharaj Nakorn Chiang Mai Hospital between January 2006 and December 2015 and divided into 2 cohorts: 1485 patients who were diagnosed from 2006 to 2009 (cohort 1: early implementation of SBCN) and 2383 patients who were diagnosed from 2010 to 2015 (cohort 2: full implementation of SBCN). Criteria to measure improved cancer waiting time (CWT) would include: time to diagnosis, time to surgery, and time to radiotherapy. The 5-year overall survival (OS) of the cohort 2 was higher than that in cohort 1, at 73.8 (72.0–75.5) compared to 71.5 (69.2–73.7) (*p*-value = 0.03).

**Conclusions:**

Reasons behind the success of project include the uniformity of care encouragement, service network development and timely access to each step of breast cancer management. The model used in SBCN could be adopted as a learning guide to improve healthcare access and outcome for breast cancer patients in low- to middle-income countries.

**Supplementary Information:**

The online version contains supplementary material available at 10.1186/s12885-021-09153-0.

## Background

The incidence of breast cancer in Thailand is higher than other types of cancer among the female population [1]. According to the estimation by the International Agency of Research on Cancer (IARC), the annual incidence of breast cancer in Thailand has steadily increased from the age-standardized incidence rate (ASR) of 17.8 per 100,000 in 1998 to 37.8 per 100,000 in 2020, and there was an approximate mortality rate with ASR of 7.6 per 100,000 in 2016 [2]. This pattern is reflected in the Northern part of the country including Chiang Mai province. Trends of breast cancer were studied in Chiang Mai population and it was found that the incidence rates increased from an ASR of 14.8 per 100,000 women-years in 1989 to 32.9 cases per 100,000 women-years in 2013 and were projected to increase to 36.7 per 100,000 women-years in 2024 [3].

The increasing incidence has raised concerns among medical practitioners and, particularly as breast cancer is a disease requiring a multimodality therapeutic approach, an integrated response is needed rather than a focus on one or few specific interventions. The difficulty also lies in the fact that there are no definitive practice guidelines in the processes of surgery, radiotherapy and systemic medications in Northern Thailand.

In Northern Thailand the lack of resources in district and provincial hospitals has been the major barrier to successful treatment of breast cancer. Limited experience and training for healthcare personnel as well as limited geographic distribution of services and resources have also been barriers. Maharaj Nakorn Chiang Mai Hospital (the supra-tertiary center) was the main center for treatment of breast cancer patients in 5 provinces in northern Thailand including Chiang Mai, Chiang Rai, Lamphun, Phayao and Mae Hong Son. As of 2005, the region has an estimated population of 4 million [4] with 6 specialist breast surgeons, 6 radiation oncologists, 6 medical oncologists, 4 pain specialists, 22 general surgeons, and only 1 radiotherapy center. Prior to 2005 all breast cancer patients from the 5 provinces who had indications for adjuvant chemotherapy and radiotherapy were referred to Maharaj Nakorn Chiang Mai Hospital. This situation posed a serious threat both to patients and healthcare personnel due to the extensive pressure on limited resources. Suandok Breast Cancer Network (SBCN), a not-for-profit organization in the university hospital which provides highly specialized medical care for patients in Chiang Mai and regionally in 5 provinces of northern Thailand, was founded in 2002 and run by volunteer breast surgeons, radiation oncologists, medical oncologists, and nurses. Our short-term goal was to decrease barriers to breast cancer management, while the long-term goal was to improve the 5-year overall survival of breast cancer patients. The objectives of this study were: 1) to assess the effectiveness of SBCN on reducing barriers to breast cancer management as a short-term goal and 2) to compare the 5-year overall survival between the 2 cohorts: early implementation of SBCN (cohort 1; 2006–2009) versus full implementation (cohort 2; 2010–2015) of SBCN as a long-term goal.

## Methods

We conducted a retrospective cohort study by examining the hospital-based data on female breast cancer patients in northern Thailand who were diagnosed from January 2006 to December 2015 at Maharaj Nakorn Chiang Mai Hospital, Faculty of Medicine Chiang Mai University.

In order to enhance breast cancer care in the locations of need, a wide range of aspects of the healthcare system is required. The areas of focus included coordination of healthcare services, a strong and expeditious referral system, collection and availability of high-quality cancer incidence and mortality data, raising awareness through national campaigns, provision of a multidisciplinary team, ensuring long-term survivor support and providing palliative-care.

The strategy was divided into 3 key operative areas which handled the identification of health service needs and issues. Each operative area was underpinned by key objectives and distinct actions.

### Key operation 1: encouraging uniformity of care (2002–2004)

The first procedure was to establish standard practice guidelines among the three treatment modalities (surgery, systemic therapy, and radiotherapy) through multidisciplinary team (MDT) conferences which were carried out from 2002 onwards. These conferences were carried out at Maharaj Nakorn Chiang Mai hospital and breast surgeons, radiation oncologists and medical oncologists were invited to discuss and agree a plan of management specific to each patient. This process is evidence-based using the guidelines recommended by the National Comprehensive Cancer Network (NCCN), the European Society for Medical Oncology (ESMO), and the American Society of Clinical Oncology (ASCO) to formulate guidance specific to Thailand. The Clinical Practice Guidelines (CPG) were developed and were recommended to the hospital network to enable the uniformity of care in this region.

### Key operation 2: development of a service network (2005–2009)

With the support from the National Health Security Office (NHSO) area 1 in 2005, the team established a partnership across 5 provincial hospitals and the supra-tertiary center and steered local services to incorporate a consistent approach across breast cancer care services. As a consequence, SBCN has been able to expand the network to 64 district hospitals/primary care clinics by 2020. The improvement has not only been in the systematized cancer care services, but coaching and regular education in breast cancer management have also been provided to SBCN members.

### Key operation 3: timely access to care (2010-present)

SBCN aimed to increase timely access to breast cancer diagnosis and treatment. The plan started with reconsidering the timeline adopted in the investigation process. The goal of a two-week timeline was set for the suspected breast cancer patient to receive the final diagnosis. In a suspected breast cancer case, mammography was performed within 2 weeks. An appointment with breast surgeons and pathologists was scheduled on the same day in a one stop service clinic for tissue biopsy and surgery was performed within 30 days of the official pathological report.

In the cases in which adjuvant radiotherapy (RT) was required, SBCN adopted and facilitated an online channel as a way to shorten the process. Network hospitals could request a RT schedule, via email in the early phases followed by on the general website of the hospital. Both regular and express appointments were made available at the discretion of the specialists, depending on the level of emergency. All necessary information for the planning of RT can be completed online. The shortened timeline has enabled patients to be able to receive RT within 6 weeks after surgery or adjuvant chemotherapy [5].

SBCN had a mission to decentralize chemotherapy treatment, therefore we worked with our network hospitals enable provision of low-level chemotherapy regimens (CMF; cyclophosphamide, methotrexate, and fluorouracil), medium-level chemotherapy regimens (Anthracycline-based) and advanced-level chemotherapy regimens (Taxane-based). The NHSO Area 1 supplied the Sterile Compounding Hood for preparing the chemotherapy injections and also supported 10-day chemotherapy nurse training workshops for network hospitals. It also constantly provided updated knowledge about breast cancer treatment to every level of healthcare personnel through lectures and workshops. The number of patients who received chemotherapy at network hospitals were recorded.

### Statistical analysis

The effectiveness of SBCN on reduction of barriers to breast cancer management as a short-term outcome was evaluated using the waiting time benchmark of the National Cancer Institute (NCI) of Thailand including: time to diagnosis, time to surgery, and time to RT using descriptive statistics. The number of patients receiving chemotherapy at the hospital network were also recorded. Five-year overall survival was studied as a long- term outcome.

All data pertinent to the diagnosis of breast cancer cases (ICD10 code C50) were extracted from Chiang Mai Cancer Registry and medical records. The alive or deceased status of the patients and the date of death were obtained from examination of the mortality data from the National Registration Department, Ministry of Interior.

Data were presented as counts and percentages for all variables. Age was categorized into young females (< 40 years), middle-aged females (40–60 years) and elderly females (> 60 years). The baseline characteristics were compared using a Chi-Square Test. Overall survival was defined as time from diagnosis of breast cancer to death from any cause using the Kaplan Meier method. Patients were censored at date of last follow up. The 5-year overall survival (OS) of SBCN patients over time, 2-year intervals between 2006 and 2015 were described and compared using the log rank test. To assess the association between time period and mortality, univariable and multivariable Cox proportional hazard models were performed. The multivariable model was adjusted by characteristics of patients including age, stage of cancer, treatment i.e., hormonal therapy, RT and chemotherapy. Statistical analyses were run using version 11 (StataCorp LP, College Station, TX, USA). All of the statistical tests employed were two-sided and with *P* < 0.05 to discern any statistically significant differences in outcomes. This study was approved by the Research Ethics Committee of Faculty of Medicine, Chiang Mai University.

## Results

According to the National Cancer Institute (NCI) of Thailand benchmark, after full setting up of the SBCN decreased barriers to breast cancer care in cancer waiting time (CWT), specifically time to diagnosis within 2 weeks in 87% of cases (*n* = 740), time to surgery within 30 days in 76% of cases (*n* = 402), time to RT within 6 weeks in 60% (*n* = 679) as shown in Table 1. We could not retrieve the data of CWT in cohort 1 patients. Number of patients receiving chemotherapy at their network hospital increased from zero in cohort 1 to 388 patients in cohort 2 (data available from 2012 to 2015).

**Table 1:** Short-term outcome of SBCN

Cancer Waiting Time (Benchmark)^a^	Benchmark accomplishment	
	Cohort 1	Cohort 2% (N)
Presentation to diagnosis (< 2 wk)	NA	87% (740)
Diagnosis to surgery (< 30 day)	NA	76% (402)
Time from surgery or chemotherapy to adjuvant RT (< 6 weeks)	NA	60% (679)

^a^National Cancer Institute of Thailand

Cohort baseline characteristics are presented in Table 2. Cohort 1 had 1485 patients, while cohort 2 included 2383 patients. Most of the patients in both cohorts were in the middle age woman band (40–60 years). Taxane-based chemotherapy regimens were prescribed in 30.9 and 9.6% of cases in cohort 2 and cohort 1 population, respectively. Use of the CMF regimen was much decreased from 13.1% in cohort 1 to 1.2% in cohort 2. Aromatase inhibitor was prescribed more frequently in cohort 2 (31.7%) than in cohort 1 (13.4%).

**Table 2:** Baseline characteristics among breast cancer subtypes, n(%)

Variables	Total	Cohort 1	Cohort 2	*P*-value^a^
Number of patients	3868	1485	2383	
Age (year)				< 0.001
< 40	445 (11.4)	204 (13.8)	241 (10.1)	
40–60	2589 (67.0)	1028 (69.2)	1561 (65.5)	
> 60	834 (21.6)	253 (17.0)	581 (24.4)	
Stage				< 0.001
I	586 (15.1)	206 (13.9)	380 (15.9)	
II	1553 (40.1)	621 (41.8)	932 (39.1)	
III	831 (21.5)	275 (18.5)	556 (23.4)	
IV	265 (6.8)	97 (6.5)	168 (7.0)	
Unknown	633 (16.4)	286 (19.2)	347 (14.6)	
Chemotherapy				< 0.001
No	1178 (30.4)	399 (26.9)	779 (32.7)	
Yes	2690 (69.5)	1086 (73.1)	1604 (67.3)	
Chemotherapy Regimens				< 0.001
CMF	161 (6.0)	142 (13.1)	19 (1.2)	
FAC/FEC/AC	1930 (71.7)	840 (77.4)	1090 (67.9)	
AC-T	599 (22.3)	104 (9.6)	495 (30.9)	
Radiotherapy				0.03
No	1794 (46.4)	722 (48.6)	1072 (45.0)	
Yes	2074 (53.6)	763 (51.4)	1311 (55.0)	
Hormonal therapy				< 0.001
No	1772 (45.8)	770 (51.8)	1002 (42.0)	
Yes	2096 (54.2)	715 (48.1)	1381 (58.0)	
Hormonal therapy agent				< 0.001
Tamoxifen	1335 (75.2)	583 (86.6)	752 (68.3)	
Aromatase Inhibitor	439 (24.8)	90 (13.4)	349 (31.7)	

*CMF* Cyclophosphamide, methotrexate, and fluorouracil; *FAC* Fluorouracil, anthracycline, and cyclophosphamide; *FEC* Fluorouracil, epirubicin, and cyclophosphamide; *AC* Anthracycline and cyclophosphamide; *AC-T* Anthracycline, cyclophosphamide, and taxane

^a^Chi-square test

**Fig. 1:**
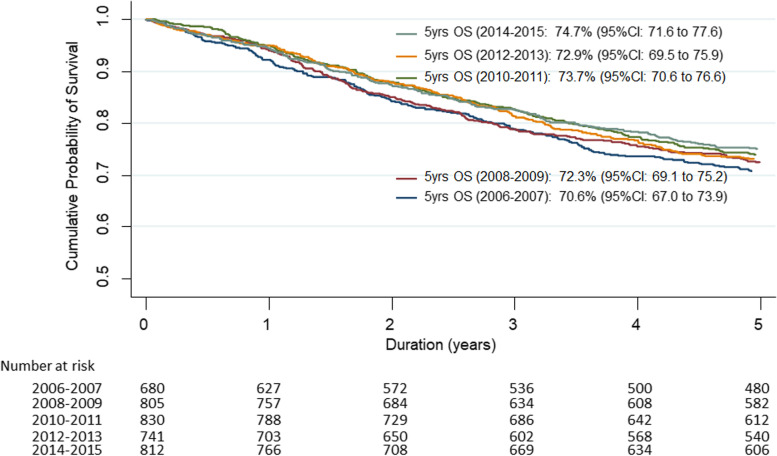
Five-year overall survival (OS) over time at 2 year intervals between 2006 and 2015

**Fig. 2:**
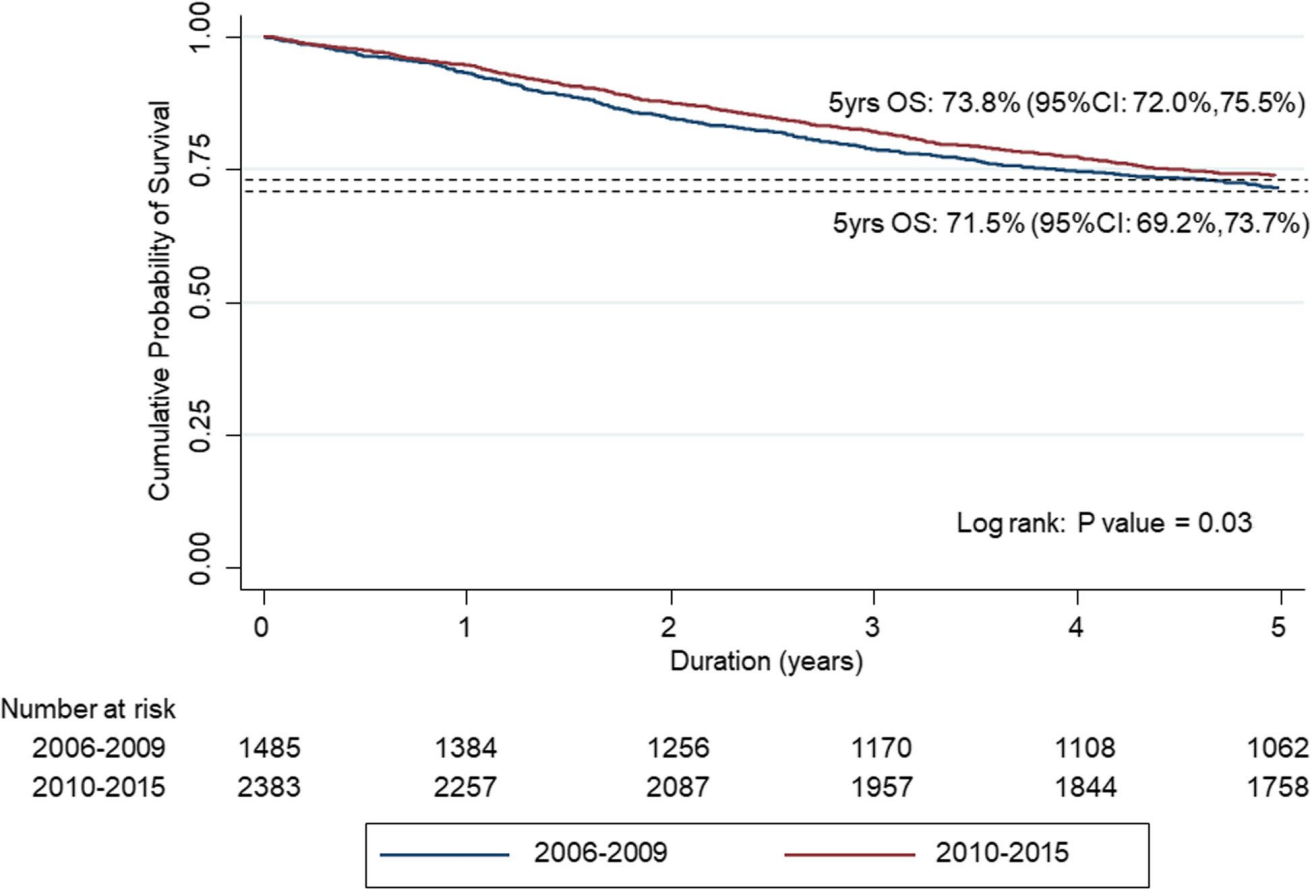
Five-year overall survival (OS) comparing between two cohorts

The 5-year overall survival (OS) of SBCN patients over time, in 2-year intervals between 2006 and 2015 is demonstrated in Fig. 1. The OS of patients diagnosed in 2006–2007, 2008–2009, 2010–2011, 2012–2013 and 2014–2015 were 70.6% (95%CI: 67.0 to 73.9), 72.3% (95%CI: 69.1 to 75.2), 73.7% (95%CI: 70.6 to 76.6), 72.9% (95%CI: 69.5 to 75.9) and 74.7% (95%CI: 71.6 to 77.6). The 5-year OS in 2006–2007 was in alignment with 2008–2009 but significantly increased in the later three periods (2010–2011, 2012–2013 and 2014–2015). Table 3 and Fig. 2 demonstrate the higher 5-year overall survival (OS) over time between cohort 1 and cohort 2 which were 71.5% (95%CI: 69.2–73.7) vs 73.8% (95%CI: 72.0–75.5), respectively (*p*-value = 0.03). The patients with early stage (I, II) in cohort 2 also had a significantly improved 5-year OS, specifically 88.6% (95%CI: 86.7–90.2) in comparison to cohort 1 85.8% (95%CI: 83.3–88.0), *p*-value = 0.02.

**Table 3:** Five-year overall survival by 2 time cohorts

	Cohort 1 OS (95% CI)	Cohort 2 OS (95% CI)	*P*-value
All stages	71.5 (69.2–73.7)	73.8 (72.0–75.5)	0.03
Early stage(I&II)	85.8 (83.3–88.0)	88.6 (86.7–90.2)	0.02
Advanced stage (III&IV)	53.5 (49.6–57.2)	55.7 (52.7–58.7)	0.21

The multivariable Cox proportional hazard regression model showed that cohort 2) was significantly associated with better OS in comparison to cohort 1 with a hazard ratio (HR) of 0.80 (*p* = < 0.001) after adjustment of the model by potential factors including stage age, in receipt of treatments included hormonal therapy, radiotherapy and chemotherapy (Table 4).

**Table 4:** Univariable and multivariable cox proportional hazard regression analysis

	Univariable cox regression (*n* = 3868)			Multivariable cox regression^a^ (*n* = 3235)		
	HR	95%CI	*P*-value	HR	95%CI	*P*-value
Cohort 1	1			1		
Cohort 2	0.89	0.80–0.99	0.03	0.80	0.70–0.90	< 0.001

^a^Multivariable model is adjusted by all variables: age, stage, hormonal therapy, radiotherapy and chemotherapy

## Discussion

The reduction of barriers to access to breast cancer care led to a satisfactory level of achievement of the short- term goal of SBCN in time to diagnosis (87%) but not when it came to time to treatment both in time to surgery (76%) and time to RT (60%). A call to action to increase the percentage of patients with timely access of surgery and RT is needed. We aim to enhance all CWT to be more than 80% in relation to the NCI of Thailand benchmark. However, the long-term outcome was achieved after full implementation of SBCN (cohort 2) with a statistically significant better 5 -year OS rate than cohort 1. However, when considering this result confounding effects from the better national health policy of Thailand need to be included. NHSO started to allow the use of Taxane in the sequential adjuvant treatment after the anthracycline-based regimen for node-positive patients in 2007 [6], non-steroidal aromatase inhibitor (AI) for adjuvant therapy in node-positive hormonal responsive postmenopausal patients in 2009 [7], and Trastuzumab for adjuvant therapy in node-positive Her2-enriched patients in 2015. As shown in Table 2 more patients in cohort 2 received high efficacy systemic medications, i.e., Taxane and AI. Although our national health policy on medication had improved the quality of cancer care, i.e., the appropriate treatment, the uniformity of care, the timeliness access to both diagnosis and the treatment was also essential. We have now provided a step by step strategic approach in the SBCN and worked to reduce the barriers at every step of the breast cancer management continuum.

The system can be implemented successfully with the cooperation of the health care personnel in the network and the continuing support from the government. However, during the early year of developing the program, these were our difficulties to face with.

One of the keys to the improvement is the adoption of online technology. As updating and implementation of technology can be relatively costly for hospitals with limited resources, initial investment should be on optimizing the basic and existing tools. SBCN allowed our network of hospitals to make an appointment for adjuvant radiotherapy through email in the earlier period followed by via the hospitals’ websites in the later period. This is a convenient, cost- and time- efficient solution for both healthcare workers and patient care.

There were some limitations in this study. First, we retrieved the death data from the National Registration Department, Ministry of Interior only. Information of the cause of death was not available, therefore we could report only the overall survival not breast cancer specific survival which may not reveal the true impact of SBCN’s work. Second, there were possible confounding effects of different national policies on breast cancer systemic treatments between the two cohorts as mentioned above. Third, the data pertinent to CWT in cohort 1 was not recorded, therefore we could not statistically compare the improvement of CWT between the two periods. Forth, due to the retrospective nature of this study could limit the standardization across the various techniques.

Most of the studies in low and middle income countries focused their aims in understanding the causes of the delayed in the interval of diagnosis and interval of care [5, 8, 9]. The cross-sectional survey from Mexico City suggested to focus on strengthening the quality of public primary care services and improving referral routes to reduce diagnosis delay [5]. Ermiah et al. interviewed 200 Libyan breast cancer patients and collected retrospective both preclinical and clinical data [8]. They concluded that the delayed in diagnosis had impacted on stage of disease and recommended to improve awareness and pay attention to the practice guideline in breast cancer [8]. The similar results were demonstrated in Iranian study [9]. They suggested to implement the interventions to lessen delays treatment and improve outcomes in this disease.

## Conclusion

To our knowledge, this is the first hospital-based study with large sample size to address the short and long term outcome of breast cancer care from healthcare professional network in Thailand and South East Asia. The SBCN model could be used as a learning guide to improve healthcare access and outcome for breast cancer patients in low- to middle-income countries.

## Supplementary Information

**Additional file 1.**

## Data Availability

The dataset used and/or analyzed during the current study is available as a supplement file.

## Abbreviations

ASR: Age-standardized incidence rate
CWT: Cancer Waiting Time
NHSO: National Health Security Office
OS: Overall survival
SBCN: Suandok Breast Cancer Network
